# Impaired spatial working memory and reduced hippocampal neuronal density in a rat model of neurocysticercosis

**DOI:** 10.3389/fncel.2023.1183322

**Published:** 2023-06-01

**Authors:** Laura E. Baquedano, Edson G. Bernal, Daniel J. Carrion, Ana D. Delgado, Cesar M. Gavidia, Daniela E. Kirwan, Robert H. Gilman, Manuela R. Verastegui

**Affiliations:** ^1^Parasitological Diagnostic Laboratory, Faculty of Sciences and Philosophy, Universidad Peruana Cayetano Heredia, Lima, Peru; ^2^School of Veterinary Medicine, Universidad Nacional Mayor de San Marcos, Lima, Peru; ^3^The Cysticercosis Working Group in Peru, Lima, Peru; ^4^Infectious Diseases Research Laboratory, Department of Cellular and Molecular Sciences, Faculty of Sciences and Philosophy, Universidad Peruana Cayetano Heredia, Lima, Peru; ^5^School of Psychology, Faculty of Philosophy and Human Sciences, Universidad Antonio Ruiz de Montoya, Lima, Peru; ^6^Infection and Immunity Research Institute, St George’s University of London, London, United Kingdom; ^7^Department of International Health, Bloomberg School of Public Health, Johns Hopkins University, Baltimore, MA, United States; ^8^Asociación Benéfica PRISMA, Lima, Peru

**Keywords:** neurocysticercosis, working memory, hippocampal density, *Taenia solium*, rat model

## Abstract

Neurocysticercosis (NCC) is the most common parasitic disease affecting the nervous system and is a leading cause of acquired epilepsy worldwide, as well as cognitive impairment, especially affecting memory. The aim of this study was to evaluate the effect of NCC on spatial working memory and its correlation with hippocampal neuronal density, in a rat model of NCC. This experimental study was conducted on female (*n* = 60) and male (*n* = 73) Holtzman rats. NCC was induced by intracranial inoculation of *T. solium* oncospheres in 14 day-old-rats. Spatial working memory was assessed using the T-maze test at 3, 6, 9, and 12 months post-inoculation, and sensorimotor evaluation was performed at 12 months post-inoculation. Hippocampal neuronal density was evaluated by immunostaining of NeuN-positive cells of the CA1 region. Of the rats inoculated with *T. solium* oncospheres, 87.2% (82/94) developed NCC. The study showed a significant decline in spatial working memory over a 1-year follow-up period in rats experimentally infected with NCC. Males showed an early decline that started at 3 months, while females demonstrated it at 9 months. Additionally, a decrease in neuronal density was observed in the hippocampus of NCC-infected rats, with a more significant reduction in rats with cysts in the hippocampus than in rats with cysts in other brain areas and control rats. This rat model of NCC provides valuable support for the relationship between neurocysticercosis and spatial working memory deficits. Further investigations are required to determine the mechanisms involved in cognitive impairment and establish the basis for future treatments.

## 1. Introduction

Neurocysticercosis (NCC) is a parasitic disease caused by *Taenia solium* larvae developing in the human central nervous system (CNS) (Garcia et al., 2014). The tapeworm *T. solium* is hosted in the human small intestine, its infectious proglottids are shed in feces, and then ingested by pigs, where the oncospheres released from the eggs, after losing their shells by pancreatic and biliary enzymes, enter the bloodstream and migrate to host tissues, developing into cysticerci. Humans can develop teniasis when eating this undercooked infected pork or can get NCC when eating food contaminated with eggs or by fecal-oral contact (Sotelo and Del Brutto, 2002).

Neurocysticercosis is a public health problem that affects urban and rural areas leading to significant morbidity and mortality. In endemic countries, it is the main cause of acquired epilepsy, and although seizures are the most common symptom of the disease, cognitive deficits and memory impairment have also been reported (Sotelo and Del Brutto, 2002; Wallin et al., 2012; Nau et al., 2018). Cognitive deterioration is far more common than previously appreciated, with reports ranging from 8.4 to 87% of patients (Forlenza, 2002; De Andrade et al., 2010; Zepeda et al., 2017), and impairments in attention and memory present in 25% (Diagana et al., 2005). Significantly reduced intelligence quotient scores in the domains of visual perception, immediate recall, analysis, synthesis, reasoning, verbal ability, memory, and spatial ability have also been reported in children with NCC (Prasad et al., 2014). However, the mechanisms underlying cognitive impairment in NCC have not been ascertained.

Different animal models have been used to study the pathogenesis of NCC using cestodes that present a cycle similar to that of *T. solium*, such as *Mesocestoides corti* and *Taenia crassiceps*. However, these models have the disadvantage of using the metacestode instead of the oncosphere as the infective stage, and also both parasites have a tendency to proliferate, which could affect the pathology studied (Arora et al., 2017). Neurocysticercosis by *T. solium* has also been evaluated using animal models such as rodents, pigs, non-human primates, among others. In the case of mice, immunosuppressed mice have been used, which limits the ability to study the host response. Monkeys have also been used, however, these animals develop a severe acute condition that usually leads to death in a few days. On the other hand, pigs have been used, although is the natural intermediate host of NCC, which represents an advantage, there are limitations regarding the handling of these animals in the laboratory, the cost, and the availability of commercial reagents to study the pathology (Sitali et al., 2022).

In our research group we have developed an animal model using Holtzman rats infected intracranially with *T. solium* oncospheres, which has allowed the development of the cysticerci in the brain (Verastegui et al., 2015). This model has the advantage that the parasite presents a morphology similar to the natural host and a pathology similar to that reported in humans (Carmen-Orozco et al., 2019, 2021; Mejia Maza et al., 2019).

On the other hand, the use of animal models has been reported to evaluate cognitive problems associated with different pathologies, such as Alzheimer’s disease, which have been widely studied using behavioral tests (Aquino et al., 2023), and to a lesser extent neuroinfectious diseases caused by viruses (Dittrich et al., 1989; Elmore et al., 2014), parasites (Parvin et al., 2016) and bacteria (Becerril-Villanueva et al., 2018), where they have also reported cognitive deterioration in addition to alterations in hippocampus, decreased learning, and short- and long-term memory loss (Tolman and Gleitman, 1949; Arime and Akiyama, 2017). In the case of memory studies in NCC, these are scarce. Recently, mouse models infected with *T. crassiceps* have been studied, where they found changes at the hippocampus and impaired memory and learning (Morales-Montor et al., 2014; López-Griego et al., 2015; Zepeda et al., 2017, 2019).

The aim of this study was to assess spatial working memory over a short period of time in rats experimentally infected with activated *T. solium* oncospheres, and to evaluate the degree of hippocampal neuronal loss associated with the presence of the parasite in the brain. Developing an animal model of NCC that allows us to study how the parasite affects the cognitive system will be an important study tool to understand the factors that are mediating this deficit and could provide information on possible therapeutic targets.

## 2. Materials and methods

### 2.1. Ethics statement

The Institutional Animal Use Ethics Committee (CIEA) of the Universidad Peruana Cayetano Heredia reviewed the study protocol and approved the experimental procedures described in this research (Approval number: No. 65259 R-016-08-17).

### 2.2. Subjects

Ten female Holtzman rats with their 11 day old pups (133 pups in total, 60 females, and 73 males) were supplied by the Universidad Peruana Cayetano Heredia, Lima, Peru. The rats were housed in the animal facilities of the School of Veterinary Medicine of the Universidad Nacional Mayor de San Marcos, Lima, Peru. The pups were weaned and separated from the mothers at 21 days of age, then randomly assigned to groups of 3 to 4 rats of the same sex. Each group was housed in a 60 L cm × 50 W cm × 22 H cm polypropylene cage lined with shavings. The rats received commercial food pellets and water *ad libitum*. To minimize stress, polypropylene tubes were placed as environmental enrichment, and the rats were monitored daily by veterinary staff. Lights were on from 06:30 h until 18:30 h and room temperature was maintained at 22–24°C.

### 2.3. Experimental design

**FIGURE 1:**
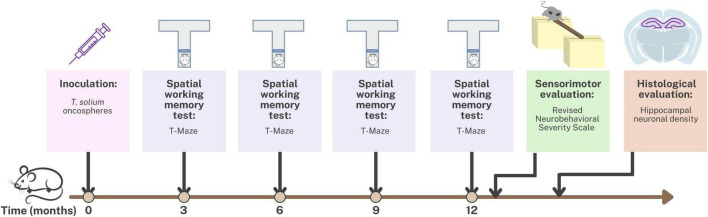
Timeline of the experimental design for the animal model of neurocysticercosis and study interventions.

The study was conducted using an animal model of Neurocysticercosis (NCC) in rats infected with *T. solium*. Figure 1 provides an overview of the experimental design used to develop this NCC model. The rats were randomly assigned to either the control group, which received 0.9% NaCl, or the experimental group, which received *T. solium* oncospheres. To induce the infection, activated oncospheres of *T. solium* were inoculated intracranially at the bregma level in 14-day-old rats (Verastegui et al., 2015). Despite the natural oral route of infection in NCC, the intracranial route was chosen for this model because rats are not the natural host of the parasite, and intracranial inoculation ensures successful infection. Previous studies using the same animal model found no differences in parasite development or CNS pathology between the oral and intracranial routes of infection. The intracranial route has the advantage of requiring only 40–500 activated oncospheres to develop the infection in the brain, while the oral route would require at least 20,000 activated oncospheres (Mejia Maza et al., 2019).

Spatial working memory testing was conducted at 3, 6, 9, and 12 months post-inoculation using the T-maze. Additionally, at 12 months post-inoculation, sensorimotor evaluation was performed using the Revised Neurobehavioral Severity Scale (NSS-R). Finally, the rats were euthanized, and hippocampal tissue was fixed and stained for histological evaluation of hippocampal neuronal density.

### 2.4. Cerebral inoculation

The Infectious Diseases Research Laboratory of Universidad Peruana Cayetano Heredia (Lima, Peru) donated the *T. solium* parasite used in the rat model (Verastegui et al., 2015). *T. solium* gravid proglottids were cut with tweezers and *T. solium* eggs were obtained. These were then centrifuged at 2500 rpm, washed in distilled water, immersed in 0.75% sodium hypochlorite solution for 10 min and washed in Roswell Park Memorial Institute (RPMI) medium to obtain oncospheres. Finally, the oncospheres were activated by the addition of artificial intestinal fluid for 45 min at 37°C and washed a further three times in RPMI medium (Verastegui et al., 2007).

Inoculation was performed using 120 activated *T. solium* oncospheres suspended in 100 μL of sterile 0.9% NaCl for the experimental group or 100 μL of sterile 0.9% NaCl for the control group. On postnatal day 14 the preparation was injected into the Bregma at 2 mm depth, releasing the oncospheres into the space between the brain parenchyma and the skull, and avoiding penetration of the needle into the parenchymal tissue itself (Verastegui et al., 2015).

### 2.5. Spatial working memory testing

**FIGURE 2:**
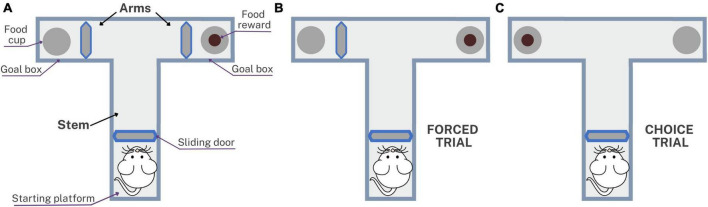
Schematic representation of the T-maze used to assess spatial working memory in rats with neurocysticercosis. **(A)** Illustration of the T-maze layout with sliding doors, starting platform, goal boxes, food cups, and food reward. The testing protocol consisted of five daily sessions, each session with one forced trial and nine choice trials. **(B)** The forced trial involved placing a reward in the open arm and blocking the other arm with the sliding door. **(C)** The choice trial involved placing a reward in the previously blocked arm, unlocking it, and allowing the rat to choose between the two open arms.

Memory was assessed using the spatial working memory test at 3, 6, 9, and 12 months post-inoculation in which the T-maze was used to evaluate the short-term memory. This method can be used to evaluate the exploratory behavior of rats, based on their willingness to explore a new environment, as described by Wenk (1998) and based on rewarded alternation version with discrete trials and enclosed arms (Wenk, 1998; Hölscher et al., 2004; d’Isa et al., 2021). Figure 2 shows the T-maze implemented to assess memory in rats with neurocysticercosis. The T-maze was constructed from black plastic in a “T” shape, consisting of a stem and two arms. Inside there were three sliding doors, a starting platform at the start of the stem, a goal box at the end of each arm, a food cup, and the food reward which was a 10 mg piece of Chocoyogurt^®^ cereal (Figure 2A). The T-maze was placed in a conditioned room with dark curtains, white background noise, and a constant level of light intensity, and the tests were started at the same time each day.

Before performing the T-maze test, a period of pre-training was carried out for 3 weeks involving animal preparation, food restriction, and habituation, to accustom the rats to the setup as described in Arime and Akiyama (2017). The test consisted of five consecutive daily sessions, each session comprising one forced trial (Figure 2B) and nine choice trials (Figure 2C). The forced trial involved placing a food reward in the open arm of the T-maze and blocking the other arm with the sliding door. The sliding door of the starting platform was lifted and the rat exited the starting platform, walked down the stem, entered the open arm, found and ate the reward, and was then returned to the starting platform. The choice trial consisted of unlocking the arm that had been blocked during the forced trial and placing a food reward in this arm. The door was then lifted and the rat was allowed to choose to enter either of the two open arms. The inter-trial interval time was 5 s, which is the time in which the rat is placed on the starting platform after a choice trial (Wenk, 1998). With repeated trials, rats would be expected to show an increasing tendency to enter a new rather than a previously visited arm. The success rate of the spatial working memory test was presented as the percentage of choices that were correct, i.e., led to the rat obtaining the food reward: (number of correct choices/number of total choices) × 100.

### 2.6. Sensorimotor evaluation

The Revised Neurobehavioral Severity Scale (NSS-R) was used to assess balance, motor coordination (including movement and postures), motor reflexes, and sensory reflexes in rats at 12 months post-inoculation. The test was performed using two large polycarbonate cages (46 L cm × 36 W cm × 20 H cm), six small polycarbonate cages (42.5 L cm × 20.5 W cm × 20 H cm), a balance beam, and cotton swabs. The NSS-R comprised ten different tests to assess general balance, landing, tail raise, dragging, righting reflex, ear reflex, eye reflex, sound reflex, tail reflex, and paw flexion reflex.

Scoring was performed using a three-point scale according to Yarnell et al. (2016), where a normal and fast response was assigned “0,” a partial or compromised response was assigned “1,” and the absence of a response was assigned “2” for each test. The final value was the sum of the 10 tests (0–20), with higher scores indicating higher neurobehavioral impairment. The test was scored by two blinded observers.

In addition, the presence of paresis or motor impairments was documented throughout the study. Animals that were unable to complete sensorimotor testing were excluded from analysis.

### 2.7. Histological evaluation

Rats were deeply anesthetized by intraperitoneal injection of Ketamine 100 mg/kg and Xylazine 20 mg/kg, then transcardially perfused with 0.9% NaCl followed by 10% formalin. The brain was dissected and post-fixed in 4% paraformaldehyde for 24 h at 4°C (Verastegui et al., 2015), then the brain tissue was embedded in paraffin and sectioned at 5 μm. Hematoxylin and eosin (H&E) staining was performed, sections were visualized at 40X on the Axio Lab A1 (Carl Zeiss, Oberkochen, Germany), captured on the AxioCam ICC camera (Carl Zeiss, Oberkochen, Germany), and processed with AxioVision software. The samples were examined by a trained pathologist.

To evaluate the degree of inflammation, an inflammation score (IS) and damage score (DS) were obtained in rats with three or fewer cysts. Rats with more than 3 cysts were not evaluated due to the requirement for evaluation of brain tissue surrounding the entire cyst. Inflammation was classified into four grades: Grade 0, minimum inflammatory infiltrate (<25 inflammatory cells/40X field); Grade 1, moderate to severe inflammatory infiltrate (≥25 inflammatory cells/40X field); Grade 2, presence of macrophages organized into a palisade; and Grade 3, presence of granulomas and/or multinucleated giant cells. The inflammation score (IS) was calculated by combining the grades and their percentage of extension: (Grade 0 ×%Extension) + (Grade 1 ×%Extension) + (Grade 2 ×%Extension) + (Grade 3 ×%Extension).

Cyst damage was classified into three grades: Grade 0, complete cyst with minimal damage to the tegument and conserved sub-tegument; Grade 1, cyst with mild damage to the sub-tegument and/or severe damage to the tegument; and Grade 2, calcified cyst, total loss of the tegument and sub-tegument, abundant presence of inflammatory cells with severe alteration of the architecture. The damage score (DS) was calculated as follows: (Grade 0 ×%Extension) + (Grade 1 ×%Extension) + (Grade 2 ×%Extension).

### 2.8. Immunostaining

Tissues were dewaxed and unmasked with buffer citrate and 0.05% tween 20, pH 6 in bath water for 20 min (ebullition). The sections were then washed with PBS, and 3% H_2_O_2_ was added for 30 min. BSA 5% protein blocking solution and 2.5% milk were added for 1 h, after which the samples were incubated with primary mouse Anti-NeuN antibody (Abcam, Ab104224, 1/1000) overnight at 4°C. The slices were then washed and incubated with HRP-labeled goat anti-mouse IgG secondary antibody (Vector Laboratories, BA-9200, 1/500) for 1 h at room temperature. Sections were revealed con DAB solution (Kit DAKO) and counterstaining was done with Harris Hematoxylin (Merck) and visualized on the Axio Lab A1 (Carl Zeiss, Germany), captured on the AxioCam ICC camera (Carl Zeiss, Oberkochen, Germany).

### 2.9. Hippocampal neuronal density

Evaluation of hippocampal neuronal density was performed by counting hippocampal neurons with Cell Counter plugin of the ImageJ software. Counting of the nuclei of each NeuN-stained neuron in the hippocampal Cornus Ammonis 1 (CA1) region in rats with a single cyst, and then the average was taken for each rat. Three fields of the hippocampus were recorded both from each hemisphere and the mean number of neurons was obtained. In addition, for rats with hippocampal cysts the neuronal density was recorded separately for the cyst side and for the side contralateral to the cyst in the hippocampal region. Also identify the disorganization of the hippocampal cells and the presence of spongiotic changes around the cyst.

### 2.10. Statistical analysis

Statistical analyses were performed using SPSS Statistics 25 (IBM, Portsmouth, UK), and Prism 9 software (GraphPad Software, San Diego, CA, USA) was used for data visualization. Normality of the data was assessed using the Kolmogorov-Smirnov test. Since the data did not exhibit normal distribution, non-parametric tests including Kruskal-Wallis, Dunn *post-hoc* tests, and Bonferroni’s test adjustment for *p*-values were performed to compare the spatial working memory test success rate, inflammation score (IS), damage score (DS), and hippocampal neuronal density between groups. The Mann-Whitney *U*-test was used to compare the NSS-R score. Data were expressed as median with interquartile range. Additionally, Spearman correlations between the spatial working memory test success rate and NSS-R score, number of cysts, IS, DS, and neuronal density were calculated. All comparisons were performed as two-tailed tests with *p*-values set as < 0.05.

## 3. Results

### 3.1. Inoculation with *T. solium* oncospheres

**FIGURE 3:**
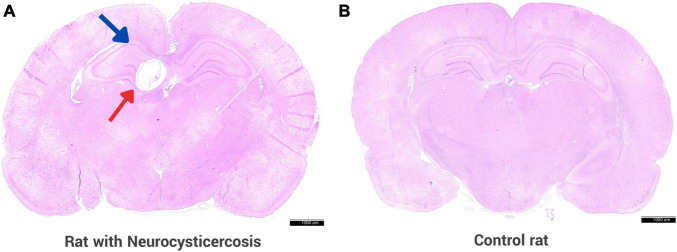
Rat animal model with neurocysticercosis. **(A)** Coronal section of a rat brain is shown, which was experimentally infected with activated *T. solium* oncospheres, resulting in the successful development of a cyst in the brain. The brain section displays a cyst (red arrow) located in the hippocampus (blue arrow). **(B)** Brain of a control rat that was inoculated with 0.9% NaCl without parasite oncospheres. Both panels were stained with H&E. Scale bar = 1000 μm.

On histological examination, cerebral cysts indicative of NCC were identified in 82/94 (87.2%) of the rats in the experimental group, 36 females and 46 males. Figure 3 shows a section of the brain of the rat from the experimental group infected with activated *Taenia solium* oncospheres, where the presence of a cyst is clearly visible in the hippocampus, unlike the control rat where there is no cyst. The remaining 12 rats did not develop any cerebral cysts and were retrospectively excluded from analysis.

### 3.2. Spatial working memory testing

**FIGURE 4:**
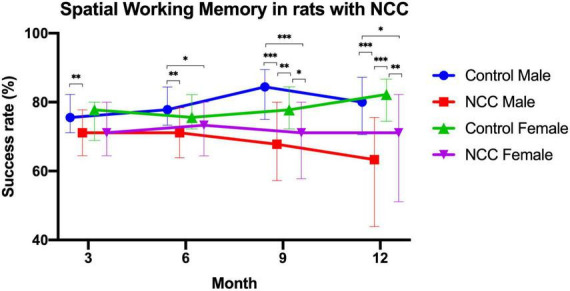
Success rate (%) of the spatial working memory test in the rat infection model for neurocysticercosis (NCC) at different time points (3, 6, 9, and 12 months post-inoculation). NCC groups consistently exhibited lower performance than the control groups, and there was a significant decline in memory success rate over time. Number of rats per group: Control male, *n* = 22; male rats with neurocysticercosis (NCC male), *n* = 42; Control female *n* = 17; and female rats with neurocysticercosis (NCC female), *n* = 35. The data are presented as median with interquartile range. *p < 0.05; ^**^p < 0.01; ^***^p < 0.001. The Kruskal-Wallis test followed by the *post hoc* Dunn test with *p* adjusted by Bonferroni’s was used to compare groups.

T-maze test was used to evaluate spatial working memory in the male rats with neurocysticercosis (NCC male), female rats with neurocysticercosis (NCC female), control male, and control female groups. The success rate (%) consistently demonstrated lower performance in rats with neurocysticercosis (NCC) compared to the control groups at 3, 6, 9, and 12 months post-inoculation, as shown in Figure 4. Figure 4 clearly illustrates a significant decline in memory success rate over time in the NCC male and NCC female groups compared to the control male and control female groups, which maintained a high success rate. Statistical analysis was conducted using Kruskal-Wallis and Dunn tests with a *p* adjusted by Bonferroni’s test.

At 3 months, male rats with neurocysticercosis (NCC) exhibited significantly lower memory success rate (median = 71%) than the male control group (median = 76%) (*p* > 0.01). At 6 months, the memory success rate was significantly higher in control male rats (median = 78%) compared to male rats with NCC (median = 71%) (*p* > 0.01) and to female rats with NCC (median = 73%) (*p* > 0.05).

At 9 months, the male rats with NCC had the lowest memory success rate (median = 68%) in comparison to the control male (median = 84%) (*p* > 0.001), and the control female (median = 78%) (*p* > 0.01). In addition, the memory success rate also was significantly lower in female rats with NCC (median = 71%) compared to control female (median = 78%) (*p* > 0.05), and control male (median = 84%) (*p* > 0.001).

At 12 months, the male rats with NCC displayed a significantly lower memory success rate (median = 63%) compared to the control male (median = 80%) (*p* > 0.001), and the control female (median = 82%) (*p* > 0.001). The female rats with NCC also demonstrated lower performance (median = 71%) compared to the control female (median = 82%) (*p* > 0.01), and the control male (median = 80%) (*p* > 0.05).

The results showed that the male rats with NCC consistently had the significant lowest memory success rate across all time points. The female rats with NCC also exhibited a lower memory success rate than the control females at all-time points, however, only were significant differences at 9 and 12 months.

### 3.3. Sensorimotor evaluation

**FIGURE 5:**
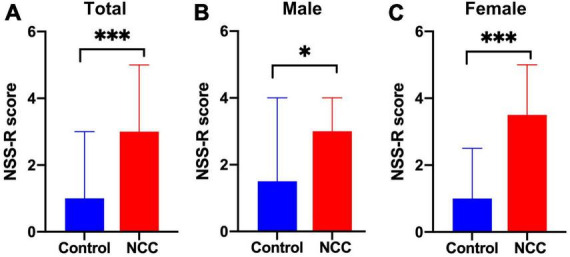
Revised neurobehavioral severity scale (NSS-R) in rats with neurocysticercosis. **(A)** The NCC group demonstrated a significantly higher NSS-R score than the control group (Mann-Whitney *U*-test: ^***^p < 0.001). Number of rats per group: Control, *n* = 39; NCC, *n* = 82. **(B)** NCC Male group exhibited a significantly higher NSS-R score than the male control group (Mann-Whitney *U* test: *p < 0.05). Number of rats per group: Control, *n* = 22; NCC, *n* = 46. **(C)** NCC Female group also displayed a significantly higher NSS-R score than the female control group (Mann-Whitney *U*-test: ^***^p < 0.001). Number of rats per group: Control, *n* = 17; NCC, *n* = 36. Data are shown as medians with interquartile range.

The NSS-R was used to evaluate the sensorimotor system, revealing a greater deficit in the NCC group compared to the control group (median = 3 vs. 1, respectively, *p* < 0.001) (Figure 5A). When males and females were evaluated separately, the male NCC group achieved higher total scores than the control male group, indicating a greater impairment of the sensorimotor system (median = 3 vs. 1.5, respectively, *p* = 0.028) (Figure 5B). Similarly, the female NCC group had a higher total NSS-R score than the control female group (median = 3.5 vs. 1, respectively, *p* < 0.001) (Figure 5C). The Mann-Whitney *U*-test was used to compare the NSS-R score between groups.

Further evaluation of the 10 individual components of the NSS-R showed that among female rats, the NCC group scored higher than the control group in the landing test (*p* = 0.017), tail raise test (*p* = 0.012), dragging test (*p* < 0.001), and righting reflex (*p* = 0.021). Among male rats, the NCC group scored higher than the control group in the dragging test (*p* = 0.003) and paw flexion reflex (*p* = 0.027) (Table 1).

**TABLE 1:** Neurological impairment measured using the revised neurobehavioral severity scale (NSS-R) at 12 months post-inoculation in male and female rats.

Test	Control male vs. NCC male	Control female vs. NCC female
	*p*-value	*p*-value
NSS-R total	0.028*	0.000*
General balance	0.596	0.649
Landing test	0.971	0.017*
Tail raise test	0.050	0.012*
Dragging test	0.003*	0.000*
Righting reflex	0.758	0.021*
Ear reflex	0.489	0.054
Eye reflex	0.696	0.110
Sound reflex	0.745	0.740
Tail reflex	0.300	0.159
Paw flexion reflex	0.027*	0.419

NSS-R, revised neurobehavioral severity scale. Comparison between groups was analyzed with Mann-Whitney U test: *p < 0.05. Number of rats per group: Control male, *n* = 22; NCC male, *n* = 46; Control female, *n* = 17; NCC female, *n* = 36.

Spearman’s rank test did not reveal any significant correlation between the total NSS-R score and the spatial working memory test. However, when analyzing the NSS-R components individually, a significant positive correlation was found between the spatial working memory test success rate and the dragging test in the male rats with NCC (rho = 0.294, *p* = 0.047). One the other hand, a significant negative correlation was observed between the spatial working memory test success rate and general balance (rho = −0.541, *p* = 0.001) and ear reflex (rho = −0.397, *p* = 0.016) in female rats with NCC.

### 3.4. Distribution of cysts

**FIGURE 6:**
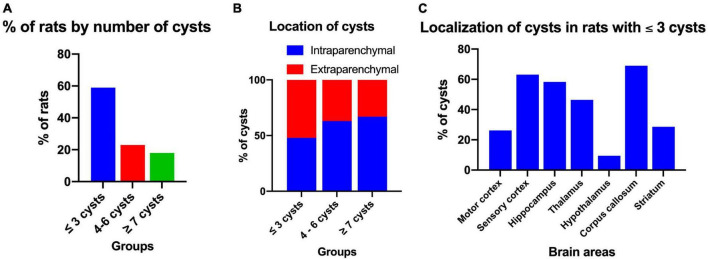
Distribution and localization of cysts in rats with NCC. **(A)** Percentage of rats according to the number of cysts in the brain, which were classified into three groups: ≤3 cysts (*n* = 45), 4–6 cysts (*n* = 18), and ≥7 cysts (*n* = 14). **(B)** Intraparenchymal and extraparenchymal localization of cysts categorized by the number of cysts in the brain. The data is presented as a percentage of cysts in each group. Number of rats per group: ≤3 cysts, *n* = 45; 4–6 cysts, *n* = 18; ≥7 cysts, *n* = 14. Number of cysts per group: ≤3 cysts, *n* = 90; 4–6 cysts, *n* = 91; ≥7 cysts, *n* = 113. **(C)** Cerebral location of intraparenchymal cysts in rats with three or fewer cysts in the brain. Data expressed in percentage of cysts. Number of rats, *n* = 42. Number of cysts, *n* = 84.

After performing the behavioral tests, the presence of cysts in the brain was evaluated. Of all the rats with NCC, it was observed that the maximum number of cysts in male rats was 10, while in female rats it was 9, with median of three cysts for both groups. However, the Mann-Whitney *U*-test showed no significant differences between the number of cysts in males and females (*p* = 0.384). Figure 6A shows the distribution of cysts into three groups: less than three cysts (≤3 cysts), 4 to 6 cysts, and more than seven cysts (≥7 cysts), and the percentage of rats per group. The majority of rats had one to three cysts (58.4%), with 57.1% in males and 60.0% in females falling into this category.

The cysts were further classified based on their location as either intraparenchymal or extraparenchymal. Figure 6B illustrates the distribution of cysts in the three evaluation groups based on the number of cysts. It is evident that in the group with less than three cysts (≤3 cysts), the minority were intraparenchymal (48%), whereas in the group with more than seven cysts (≥7 cysts), the majority were intraparenchymal (67%). The analysis with the Spearman correlation test provided a significant correlation between the success rate of the spatial working memory test and the number of cysts per rat (rho = −0.651, *p* < 0.001) indicating that the greater the number of cysts there is a negative relationship with memory.

For histological evaluation, rats with ≤3 cysts and intraparenchymal localization were selected. Cysts were identified in various regions of the brain, including the hippocampus, sensory cortex, motor cortex, basal ganglia, thalamus, hypothalamus, and corpus callosum, with the highest percentage in the sensory cortex, corpus callosum and hippocampus (66.0, 66.0, and 57.4%, respectively). In most cases, the same cyst was found to extend across multiple brain area (Figure 6C).

Then the inflammation score (IS) and damage score (DS) were assessed in three groups based on the number of cysts present: rats with one cyst (*n* = 12), rats with two cysts (*n* = 10), and rats with three cysts (*n* = 9), with a total of 12 cysts, 20 cysts, and 27 cysts evaluated, respectively. Since the data did not follow a normal distribution, the medians of the groups were compared. The results showed that the group of rats with one cyst had the highest inflammation score (IS) (median = 30), followed by the group of rats with three cysts (median = 15) and group of rats with two cysts (median = 10). In contrast, all three groups had a median damage score (DS) equal to 0. The Kruskal-Wallis test indicated that there were no significant differences in either the IS [H (2) = 1.44, *p* > 0.05] or the DS [H (2) = 3.95, *p* > 0.05]. Furthermore, when male and female rats were compared using the Kruskal-Wallis test, there were no significant differences in either the IS (*p* = 0.448) or the DS (*p* = 0.185). To examine the relationship between the inflammation score (IS) and spatial working memory test success rate, Spearman correlation was performed, but no significant correlation was found (rho = 0.03, *p* = 0.85). However, there was a borderline negative correlation between the damage score (DS) and spatial working memory test success rate (rho = −0.33, *p* = 0.05).

### 3.5. Impact of cyst location on spatial working memory

**FIGURE 7:**
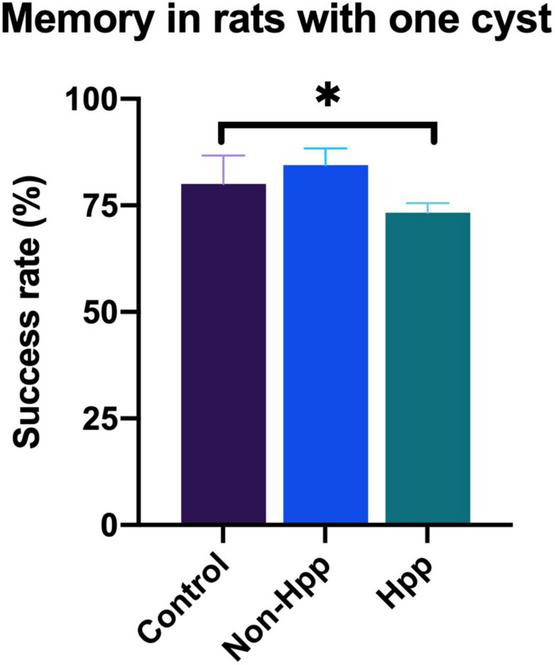
Spatial working memory testing in rats with a single cyst in the brain. Rats with a hippocampal cyst exhibited a lower success rate of spatial working memory compared to those with a cyst located outside the hippocampus and control rats. The number of rats evaluated in each group were: Hpp (Cyst in the hippocampus), *n* = 7; Non-Hpp (Cyst outside the hippocampus), *n* = 8; C (Control), *n* = 39. The data is expressed as median with interquartile range and statistical significance was determined using Kruskal-Wallis and *post hoc* Dunn test (*p < 0.05).

Rats with a single cyst were divided into two groups depending upon whether the cyst was located within to the hippocampus (Hpp) or external to the hippocampus (Non-Hpp) in order to compare the differences in memory between these groups. The spatial working memory test in rats with one cyst is shown in Figure 7. Rats with a cyst in the hippocampus showed lower success rate (Hpp, median = 73.3) compared to trats with a non-hippocampal cyst (Non-Hpp, median = 84.4) and control rats (Median = 80). A Kruskal-Wallis test revealed a significant difference among the groups [H (2) = 5.8, *p* < 0.05]. Subsequently, a Dunn *post-hoc* tests were performed with *p* adjusted by the Bonferroni’s test. A significant difference in memory performance was observed between rats with a hippocampal cyst and control rats (*p* = 0.02). No significant differences were found between rats with a single non-hippocampal cyst compared to control rats (*p* = 0.39), or between rats with a single non-hippocampal cyst compared to those with a hippocampal cyst (*p* = 0.06).

### 3.6. Hippocampal neuronal density

**FIGURE 8:**
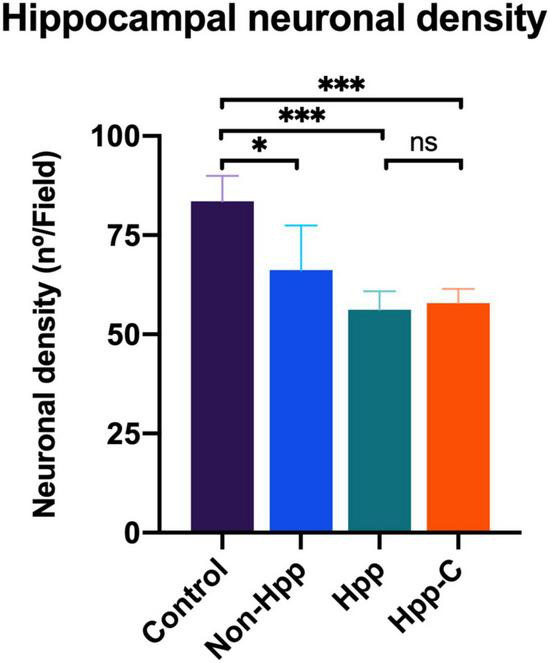
Hippocampal neuronal density in rats with single cyst in the brain. The results reveal a significant reduction in the density of hippocampal neurons in rats with cyst located in the hippocampus as well as in their contralateral side. Moreover, rats with cysts located outside the hippocampus also show a decrease in neuronal density. The number of rats evaluated in each group were: Control, *n* = 9; Non-Hpp (cyst outside the hippocampus), *n* = 5; Hpp (cyst in hippocampus), *n* = 4; Hpp-C (contralateral side of cyst in hippocampus), *n* = 4. The data is presented as the median with interquartile range and statistical significance was determined using Kruskal-Wallis test and *post hoc* Dunn test (*p < 0.05, ^***^p < 0.001, *ns* = non-significant *p*).

Neuronal density in the hippocampal CA1 region of rats with a single cyst was assessed, and only specimens with sufficiently high quality tissue sections to enable quantification of neurons were included in the analysis. Four groups were evaluated, including hippocampus from rats with a single hippocampal cyst (Hpp, *n* = 4), contralateral hippocampus from rats with a single hippocampal cyst (Hpp-C, *n* = 4), hippocampus from rats with a single extra-hippocampal cyst (Non-Hpp, *n* = 5) and hippocampus from control rats (*n* = 9). The Figure 8 depicts the hippocampal neuronal density, which was notably lower in both rats with hippocampal cysts (Hpp, median = 56.2 n°/field) and their contralateral hippocampus (Hpp-C, median = 57.8 n°/field), followed by rats with non-hippocampal cysts (Non-Hpp, median = 66.2 n°/field), and a high hippocampal density in the control rats (Median = 83.5 n°/field). Then the Kruskal-Wallis test revealed a highly significant difference between the groups [H (3) = 14.67, *p* < 0.01], and the Dunn *post-hoc* tests were performed with *p* adjusted by Bonferroni’s test to compare and find the differences between groups. Highly significant differences were found between rats with single hippocampal cyst and control (*p* < 0.001) and between contralateral hippocampus from rats with single hippocampal cyst and control (*p* < 0.001). Additionally, there were differences between rats with single extra-hippocampal cyst and control (*p* < 0.05). Interestingly, among rats with a single cyst in the hippocampus, the low neuronal density did not show significant differences in the hippocampus on the side where the hippocampal cyst was found (Hpp) vs. its contralateral side (Hpp-C) (*p* = 0.87). Spearman’s correlation was assessed to find the relationship between neuronal density and results of spatial memory testing, but no significant correlation was found (rho = 0.37, *p* = 0.135).

**FIGURE 9:**
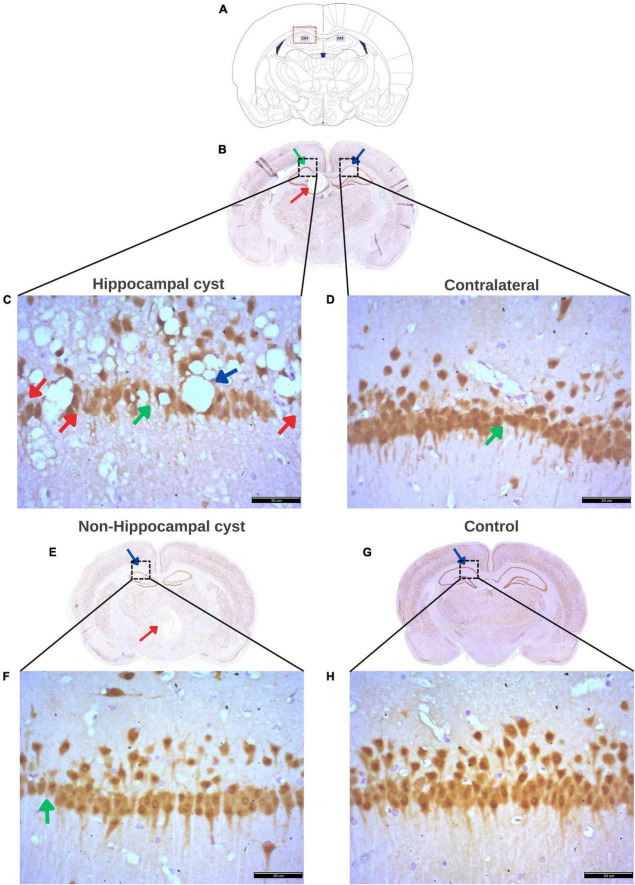
Histological findings in the hippocampus of rats with a cyst. **(A)** Schematic representation of the Cornus Ammonis 1 (CA1) region of the hippocampus where evaluation was performed. **(B)** Rat brain with a cyst in the hippocampus (red arrow) and indicating the CA1 region (green arrow) where neuronal density was evaluated, and the contralateral CA1 region (blue arrow) where the contralateral density was evaluated. **(C)** Decreased density of hippocampal neurons (red arrows), increased distance between neurons (green arrow), numerous and large vacuoles (spongy changes) (blue arrow), and marked disorganization of hippocampal neurons in rats with a single cyst located in the hippocampus. **(D)** Increased distance between neurons (green arrow), low number of hippocampal neurons, and neuronal disorganization on the contralateral side of the hippocampal cyst. **(E)** Rat brain with a cyst in an area other than the hippocampus (red arrow) with a blue arrow indicating CA1 where the assessment of hippocampal density was made. **(F)** Decreased hippocampal neurons and marked increase in distance between neurons (green arrow) in the group of rats with a single cyst located in any area other than the hippocampus, that is, non-hippocampal cysts. **(G)** Control rat brain with CA1 area (blue arrow) for hippocampal evaluation. **(H)** Ordered hippocampal neurons without vacuoles and with little distance between neurons and a greater number of neurons in the hippocampus in rats without cysts (control group). All panels **(B–H)** were stained with NeuN. Scale bar = 50 μm, 40× magnification.

Following the NeuN staining to identify and quantify neurons in the hippocampus, the histopathological findings in each group were described. Figure 9 displays the regions evaluated for hippocampal density, along with notable histological changes. Rats with a hippocampal cyst showed a significant reduction in hippocampal neuronal density, accompanied by increased neuronal distance and a large number of vacuoles (spongy changes) with marked neuronal disorganization. The contralateral side also exhibited fewer neurons, greater distance between neurons, and some disorganization. In the case of rats with cysts in regions outside the hippocampus, a decrease in the number of neurons and an increase in the distance between them was observed.

## 4. Discussion

This study demonstrated a significant reduction in spatial working memory over 1 year of follow-up in rats experimentally infected with NCC. Male rats demonstrated this impairment at an earlier age than females. The study also demonstrated a reduction in hippocampal neuronal density in rats with NCC. This occurred even when the cyst was located in an area other than the hippocampus, and for rats with hippocampal cysts neuronal density was decreased on both the side with the cyst and the contralateral side. Decreased hippocampal neuronal density was also associated with impairments in spatial memory.

The hippocampus is a highly plastic structure due in part to the presence of adult neurogenesis in the dentate gyrus, fluctuations in dendritic spine/synapse density, dendritic arborization (McEwen, 2018), and electrophysiological plasticity with long-term potentiation and long-term depression (Yagi and Galea, 2019), which makes it susceptible to damage and alterations in cognition (Bliss and Collingridge, 1993). It plays an important role in spatial learning, temporal learning, and memory paradigms (Morris et al., 1982; Logue et al., 1997; Solari and Hangya, 2018). An association between the structure and function of the hippocampus and spatial orientation has been identified in rodents (Wood and Dudchenko, 2003) where direct damage to the hippocampus leads to memory deficits. This agrees with our findings where direct damage is observed in the hippocampus due to the cyst, where we observed a decrease in neurons, an increase in the distance between neurons, an alteration in the organization of neurons, and the presence of spongy change as the vacuolization on the hippocampus in rats with a hippocampal cyst associated with impaired spatial working memory. However, we also observed similar histopathologic features in the contralateral hippocampus of these same rats with hippocampal cyst, lesions such as increased neuronal distance, low number of hippocampal neurons, and neuronal disorganization, but with less severity. This finding suggests that the hippocampus would not only be altered by direct damage but also by distant damage in the contralateral hippocampus where the cyst was located, similar to what was mentioned in another study where they suggest that the *T. solium* cyst is able to exert pathological changes in distant brain regions (Carmen-Orozco et al., 2021).

Moreover, in our data a decrease in hippocampal neuronal density was also observed in rats with cysts outside the hippocampus. This may be mediated via networks between the hippocampus and cortical regions of the brain, and may be responsible for alterations in fronto-parieto-temporal networks related to intellectual functioning which have been observed in NCC (Ramirez-Bermudez et al., 2005; Kumar et al., 2013). Memory deficit is multifactorial, and different pathways can cause alterations in the structure and function of the hippocampus (Kumar et al., 2013). Widespread apoptosis of hippocampal cells and surrounding neurons has been seen in *Taenia crassiceps* (Zepeda et al., 2011, 2019) where it is suggest that metacestode factor may diffuse through the bloodstream or tissue and induce widespread apoptosis of the hippocampus and surrounding cells, leading to a reduction in neuronal density and thus causing memory deficits (Zepeda et al., 2017, 2019). Further studies are needed to investigate whether a similar mechanism occurs in humans infected with *T. solium* (Del Brutto et al., 2016).

Our findings that rats with NCC develop a significant impairment in spatial working memory echoes effects described in patients with NCC. Mild cognitive deficits and a marked decrease in quality of life have been reported in newly diagnosed adults and children with NCC (Sotelo and Del Brutto, 2002; Wallin et al., 2012; Nau et al., 2018). Cognitive deterioration, in particular, is far more common than previously recognized (Zepeda et al., 2017): signs of psychiatric illness and cognitive decline have been reported in 65.8 and 87% of patients with NCC, impaired attention in 100% of patients, and impaired memory in 25% of the subjects examined (Forlenza, 2002; De Andrade et al., 2010).

In an Ecuadorian urban Andean community, they evaluated cognitive impairment with the Mini mental state test (MMS), and made the diagnosis of cysticercosis with a computed tomography (CT) scan and with an enzyme-linked immunoelectrotransfer blot (EITB) assay, of which 21% tested positive for cysticercosis out of a total of 214 people over the age of 59 years, and they found that the prevalence of cognitive impairment was 23.5% (8 cases) in adults over 75 years (*n* = 34) (Diagana et al., 2005). In another study, children with NCC were found to have significantly decreased intelligence quotient (IQ) scores, particularly affecting the domains of visual perception, immediate recall, analysis, synthesis, reasoning, verbal ability, memory, and spatial ability (Prasad et al., 2014). However, the mechanisms that underlie cognitive deterioration in NCC have still not been identified. Possibilities include mechanisms leading to axonal swelling, which has been associated with memory loss and cognitive deficits (Pandey et al., 2021). Studies using the rat NCC model have also found that NCC can cause dysfunctional autophagy (personal communication), and is associated with memory and cognitive deficits (Zhao et al., 2015). Further work in this area is needed.

Motor and sensory neurological deficits in patients with NCC have also been reported, for example, a patient with NCC who presented acute left-sided leg, arm, and facial weakness. The authors inferred that the vasogenic edema surrounding the scolex most likely accounted for the acute onset of the neurological deficit (Deng et al., 2019). Another case report described a patient with focal neurological features including hemiparesis, altered consciousness, memory disorder, and gait instability in whom MRI showed a multilobulated fronto-parieto-temporal cystic lesion which displaced adjacent brain elements, caused midline shift, and collapsed the ipsilateral lateral ventricle, compatible with a giant intraparenchymal cyst in vesicular stage (Brito-Núñez et al., 2019).

Our results of an earlier onset of memory deficits in male compared to female rats may be due to hormonal differences. The hippocampus contains sex hormone receptors such as androgen receptors, and α, β, and G-coupled protein estrogen receptors. The CA3 and CA4 regions of the hippocampus in adult female rats contain more estrogen receptor-β compared to males (Zhang et al., 2002) whereas male rats have higher levels of androgen receptors in the CA1 region and dentate gyrus compared to females (Feng et al., 2010). Moreover, the distribution of α and β estrogen receptors and progesterone receptors in females varies considerably according to the estrous cycle (Mitterling et al., 2010).

Sheppard et al. (2019) described the importance of estrogen and its role in injury tolerance and neuronal plasticity, modulating and mediating synaptic spines and synapse formation, and neurogenesis in hippocampal formation. Estradiol has been shown to attenuate cell death after ischemic damage and to promote neuronal survival and tissue integrity (Villa et al., 2016). These neuroprotective effects occur due to estrogen’s action on microglia, particularly on α estrogen receptors which are implicated in cerebral infarction (Sheppard et al., 2019).

However, the neuroprotective function of estrogen is time-critical. We found that at 12 months post-inoculation the females exhibited more marked memory deficits than males. This critical period of estrogen neuroprotection is described in a study in which the administration of estrogens to women immediately after menopause led to a reduction in cognitive impairment, whereas receipt of estrogens long after menopause increased the risk of cognitive impairment (Azcoitia et al., 2019). Sex, age, and the estrous cycle affect mRNA and protein expression of these hormone receptors in the hippocampus (Yagi and Galea, 2019). Understanding how structural plasticity in the hippocampus is affected by estrogens and how this is influenced by other factors is critical for the development of future therapies for cognitive decline (Sheppard et al., 2019).

This study represents a first step toward understanding the impact of *T. solium* on memory. However, there are certain limitations to consider. Firstly, although a large number of animals were inoculated and despite standardization of the inoculum, the numbers of cysts that developed varied due to the unpredictable nature of cyst development. This rendered it difficult to perform a homogeneous analysis since histological examination for inflammation and damage evaluation required an intact cyst with complete surrounding brain tissue, which reduced the sample size for this component of the analyses. In addition, many of the rats had multiple extraparenchymal and intraparenchymal cysts that could lead to mass effect and thereby confound the data. Conversely, the selection of the adapted maze for this study was advantageous as it is not very stressful to the animals and is easy to set up. Although only one behavior was assessed in this study, future studies may explore additional behaviors.

## 5. Conclusion

In conclusion, this study demonstrated a pronounced decline in spatial working memory as well as a decrease in hippocampal cell density in rats with NCC. These findings occurred in rats with cysts in both the hippocampus and in non-hippocampal regions of the brain. This NCC rat model is effective, and will allow us to evaluate other factors including seizures pattern, cytokines profile, and a more detailed evaluation of hippocampal damage in the future. Further investigation is necessary to understand which factors mediate the observed cognitive deficits, to clarify which treatment interventions may be effective to mitigate the effects of NCC on cognitive function, and to establish whether any resulting cognitive deficit is fully or partially reversible.

## Data availability statement

The raw data supporting the conclusions of this article will be made available by the authors, without undue reservation.

## Ethics statement

This animal study was reviewed and approved by the Institutional Animal Use Ethics Committee (CIEA) of the Universidad Peruana Cayetano Heredia reviewed the study protocol and approved the experimental procedures described in this research (Approval number: 65259 R-016-08-17).

## Author contributions

LB involved in the study design, data analysis, and writing of the manuscript. EB performed the immunohistological experiments and the interpretation. DC conducted the literature search and implemented and validated the method. AD contributed to conducting the behavioral experiments. CG, DK, and RG contributed to the interpretation and writing of the results. MV conceived the project idea and supervised the project. All authors contributed to the manuscript revision, read, and approved the submitted version.
